# Precise control of ion channel and gap junction expression is required for patterning of the regenerating axolotl limb

**DOI:** 10.1387/ijdb.200114jw

**Published:** 2020

**Authors:** KONSTANTINOS SOUSOUNIS, BURCU ERDOGAN, MICHAEL LEVIN, JESSICA L. WHITED

**Affiliations:** 1 Department of Stem Cell and Regenerative Biology, Harvard University, Cambridge, MA; 2 The Allen Discovery Center at Tufts University, Medford, MA; 3 Wyss Institute for Biologically Inspired Engineering, Harvard University, Boston, MA; 4 The Harvard Stem Cell Institute, Cambridge, MA, USA

**Keywords:** Axolotl, Regeneration, digit patterning, gap junction, ion channel

## Abstract

Axolotls and other salamanders have the capacity to regenerate lost tissue after an amputation or injury. Growth and morphogenesis are coordinated within cell groups in many contexts by the interplay of transcriptional networks and biophysical properties such as ion flows and voltage gradients. It is not, however, known whether regulators of a cell’s ionic state are involved in limb patterning at later stages of regeneration. Here we manipulated expression and activities of ion channels and gap junctions *in vivo*, in axolotl limb blastema cells. Limb amputations followed by retroviral infections were performed to drive expression of a human gap junction protein Connexin 26 (Cx26), potassium (Kir2.1-Y242F and Kv1.5) and sodium (NeoNav1.5) ion channel proteins along with EGFP control. Skeletal preparation revealed that overexpressing Cx26 caused syndactyly, while overexpression of ion channel proteins resulted in digit loss and structural abnormalities compared to EGFP expressing control limbs. Additionally, we showed that exposing limbs to the gap junction inhibitor lindane during the regeneration process caused digit loss. Our data reveal that manipulating native ion channel and gap junction function in blastema cells results in patterning defects involving the number and structure of the regenerated digits. Gap junctions and ion channels have been shown to mediate ion flows that control the endogenous voltage gradients which are tightly associated with the regulation of gene expression, cell cycle progression, migration, and other cellular behaviors. Therefore, we postulate that mis-expression of these channels may have disturbed this regulation causing uncoordinated cell behavior which results in morphological defects.

## Introduction

How an axolotl regenerates its amputated limb to the exactly correct shape and size has been an intriguing question to scientists studying regeneration. How do large numbers of cells coordinate their activities to form and repair specific large-scale structures? A blastema, which houses a population of highly-proliferative cells and forms at the stump upon amputation, coordinates the establishment of the missing tissue part. Cell proliferation, differentiation and migration are essential cellular activities for replacing the missing tissue and drivers of these cellular events are mostly studied at the gene expression and biochemical signaling level. In addition to changes in gene expression levels and biochemical signaling, cells can also exploit the biophysics of ion-mediated electrical signaling to communicate and establish tissue-level patterning after injury. Classical data revealed the importance of endogenous trans-epithelial electric fields for limb development and regeneration. For example, following limb amputation in newts, a current efflux from the stump occurs early in the regenerative process, and experimentally reversing this causes regenerative failure (Borgens *et al.*, 1977, Borgens *et al.*, 1979b, Borgens *et al.*, 1979c). More recent work has focused on the roles of endogenous gradients of cellular resting potential during morphogenesis (Whited and Levin, 2019, McLaughlin and Levin, 2018).

Endogenous spatio-temporal patterns of resting potentials (voltage across a cell’s membrane), are established and maintained through the function of ion pumps, channels, and pores located within the cell surface. Importantly, these biophysical properties are not only critical for neural function but are exploited by all cell types in the body for regulation of growth and form (Bates, 2015, Levin and Martyniuk, 2018, Funk, 2013). Thus, modulation of endogenous gradients of cellular resting potential has been used for induction or augmentation of regeneration in amphibian (Adams *et al.*, 2007, Tseng *et al.*, 2010, Adams *et al.*, 2013, Borgens *et al.*, 1979a) and even mammalian (Smith, 1981, Leppik *et al.*, 2015) appendages.

**Fig. 1.**
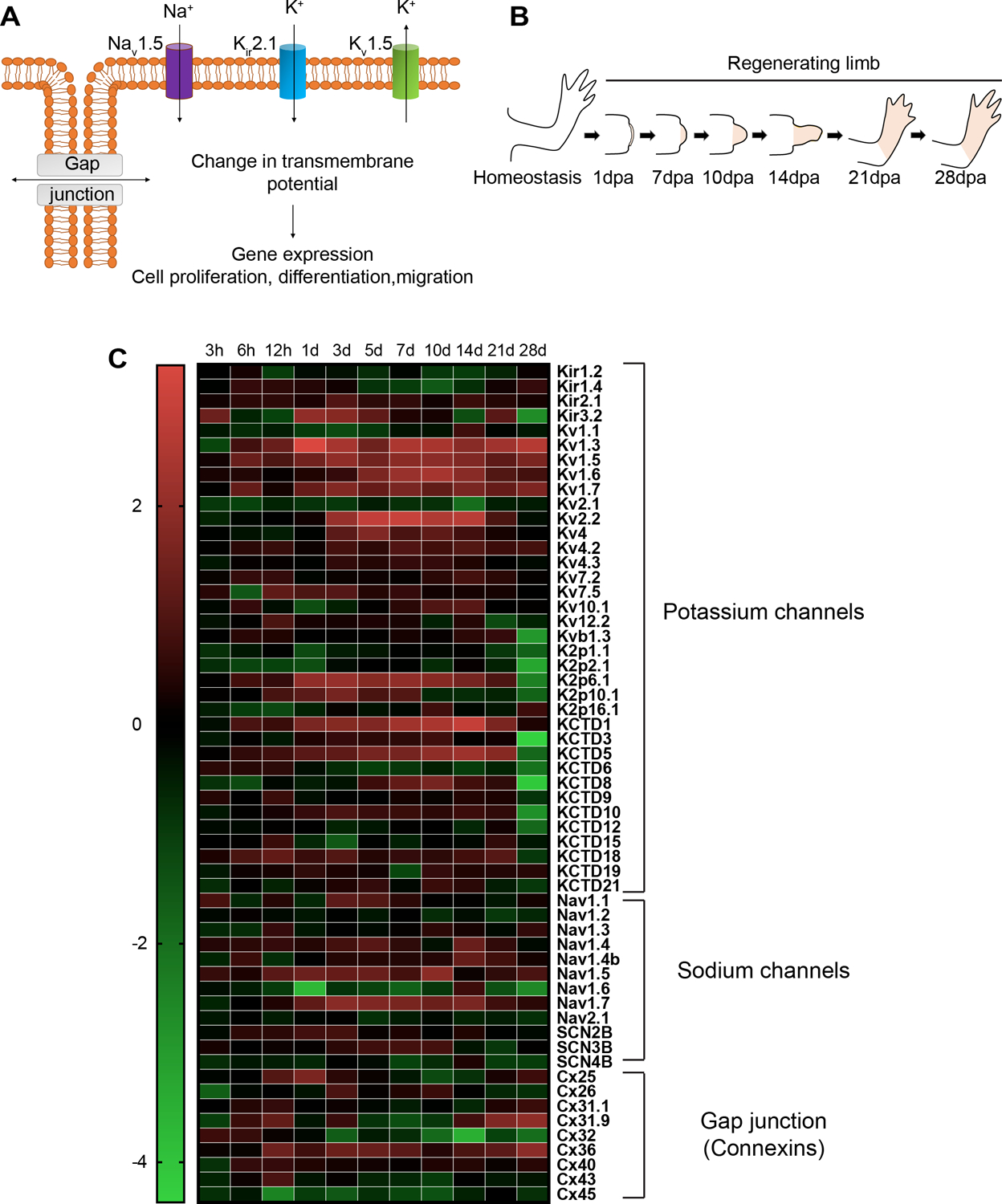
Ion channel and gap junction isoforms are transcribed in regenerating forelimb tissues. **(A)** *Schematic representation of sodium (Na^+^), potassium (K^+^) ion channels and gap junctions located on the cell membrane mediating ion exchange to induce transmembrane potential to activate downstream cellular activities*. **(B)** *Cartoon showing the stages of forelimb regeneration*. **(C)** *Heatmap of the log2 ratios ofTPMs (transcripts per million) of transcripts encoding potassium channels, sodium channels, and gap junction forming protein connexins at different time points during forelimb regeneration relative to homeostasis (0 h)*.

Bioelectric states are fundamentally tissue-level properties, propagating across groups of cells, as cells can be connected to one another via gap junctions – electrical synapse channels that connect neighboring cell membranes via protein complexes and allow direct diffusion of small molecules and ions (Palacios-Prado and Bukauskas, 2009) (Fig. 1A). Overall, ion channels set the resting potential of specific cells, and gap junctions regulate the topology of tissue-level bioelectric networks by regulating the boundaries between cell fields with distinct membrane voltage potential (Vmem) states (Levin, 2017).

Changes in membrane potential can be an instructive signal transduced to downstream effectors to initiate important cell functions like gene expression activity, cell cycle entry or differentiation (Funk, 2015, Mathews and Levin, 2018), all of which are activities relied on during regeneration. Altering the native distribution of the ion flow and hence membrane potential can specifically alter tissue patterning and large-scale anatomy. For example, kcnk5b, a two-pore potassium channel and gap junction protein connexin43 have been implicated in regulating fin growth during zebrafish fin development and regeneration by inducing cell proliferation (Perathoner *et al.*, 2014, Hoptak-Solga *et al.*, 2008). Another study showed that hyperpolarization of non-eye cells causes ectopic eye formation during *Xenopus laevis* development by inducing expression of eye patterning genes (Pai *et al.*, 2012b). The precisely established morphogen gradients which play an instructive role during tissue patterning can also be affected by disturbed ion flow. For example, studies in fly wing identified that potassium channel Kir2.1 regulates release of Dpp (*Drosophila* ortholog of Bmp), hence affecting wing disc morphogenesis during development. This regulation was shown to be linked to intracellular calcium oscillations in which Kir2.1 inhibition decreases duration and amplitude of calcium transients resulting in impaired Dpp release (Dahal *et al.*, 2017).

Interplay between ionic state and morphogen gradients is not unidirectional. Shh- and Wnt-induced activation of connexin43 expression facilitates gap junctional communication which allows tissue-wide long-range Ca++ oscillations that coordinate cell movement during feather bud growth and patterning (Li A. *et al.*, 2018). Similarly, in *Xenopus* brain patterning and planarian head-tail axial patterning, bioelectric circuits work in feedback loops with Notch and Wnt signaling respectively to regulate growth and form (Pai *et al.*, 2015a, Beane *et al.*, 2013, Durant *et al.*, 2019).

Human channelopathies due to mis-regulated ion channels have also been implicated in morphological defects, some of which are associated with digit formation. For instance, Andersen-Tawil Syndrome in which mutations in the Kir2.1 potassium channel results in craniofacial (Adams *et al.*, 2016a) and limb abnormalities that include defects like digit shortening and fusion (syndactyly) (Plaster *et al.*, 2001). Examples of such chanellopathies with limb and digit formation defects can be extended to other ion channels too. For instance, mutations in Cav1.2 channels in Timothy syndrome (Splawski *et al.*, 2004), sodium leak channel mutations in Freeman-Sheldon syndrome (Chong *et al.*, 2015) and mutations in voltage gated potassium channel KCNH1 (Kv10.1) in Temple-Baraitser syndrome (Simons *et al.*, 2015) are examples of channelopathies due to mis-regulated ion channels. However, the role of ion channels in patterning the regenerating limb has not been previously characterized. Therefore, we reasoned that changes in membrane potential, in individual cells or across fields of cells (Fig. 1A)., could be an important feature of successful axolotl limb regeneration (Fig. 1B).

Once a blastema has formed, communication between blastema cells is likely to be critical for establishing the anatomical pre-pattern of the limb and for coupling other cellular events, such as differentiation and the limb ultimate pattern. To test the hypothesis that electrogenic protein activity is important for regenerative patterning, we first datamined expression of ion channels and gap junctions from an existing transcriptomics dataset derived from homeostatic (intact, uninjured) and regenerating axolotl limbs (Stewart *et al.*, 2013) and found a dynamic regulation of these genes during blastema formation and patterning. We then sought to functionally manipulate the native membrane potential in blastema cells by overexpressing human ion channel proteins Kir2.1-Y242F, Kv1.5, NeoNav1.5 or gap junction protein connexin26 (Cx26) - constructs previously characterized to be efficient targets to specifically modify endogenous bioelectrical states in a number of developmental and regenerative systems (Pai *et al.*, 2012a, Pai *et al.*, 2015a, Blackiston *et al.*, 2011, Adams *et al.*, 2007). Kir2.1-Y242F mutants, constitutively active forms of Kir2.1 that replace tyrosine 242 with phenylalanine, were shown to cause hyperpolarization of myoblasts. In turn, this was shown to trigger expression of transcription factors to initiate myogenic differentiation (Hinard *et al.*, 2008). Kv1.5 injected in *Xenopus* embryos was shown to induce ectopic eye formation due to hyperpolarization. In some circumstances, embryos injected with NeoNav1.5 mRNA also induced ectopic eye information. Furthermore, formation of ectopic eyes following alteration in membrane potential was shown to be due to the changes in the expression levels of eye development markers (Pai *et al.*, 2012b). The gap junction protein Cx26 lacks intracellular regulatory regions and thereby facilitates constitutively permeable gap junction formation, wiping out endogenous boundaries between neighboring compartments, such as the Left and Right sides during development of laterality (Levin and Mercola, 1998).

Analysis of skeletal morphology demonstrated that limbs overexpressing ion-channels Kir2.1(Y242F), Kv1.5, NeoNav1.5, or gap junction protein Cx26 during regeneration developed defects associated with number and morphology of the digits. In addition to overexpression data, acute inhibition of gap-junction function via Lindane treatment resulted in missing carpals and digits compared to DMSO controls. Our findings suggest that manipulating the expression levels of ion channels or gap junction proteins, which are regulators of endogenous gradients of cellular resting potential, in blastema cells alters the morphology of regenerated limb.

## Results

### Gap junction and ion channel proteins are dynamically regulated at different stages of regeneration

To address whether gap junction and ion channel proteins may be important during limb regeneration, we investigated their expression profiles at different stages of regeneration. Several datasets have defined the global gene expression of bulk tissue derived from homeostatic (intact, uninjured) as well as regenerating axolotl limbs (Stewart *et al.*, 2013). The analysis of these transcriptomic data confirmed that a diverse array of ion channel and gap junction isoforms are transcribed in regenerating forelimb tissues (Fig. 1C). According to the gene ontology (GO) analysis performed by Stewart *et al.*, with regenerating limb tissue collected over time, GO terms for ion homeostasis are enriched between 12 h – 1 day period of regeneration while genes representing cation homeostasis enriched between 1– 3 days and calcium ion binding enriched between 1–10 days (Stewart *et al.*, 2013).

Enrichment of GO terms related to tissue development and morphogenesis (such as limb morphogenesis, bone development, skeletal system morphogenesis) spans the period between 7 d-14 d of the regeneration process (Stewart *et al.*, 2013). We therefore wished to examine the consequences of challenging the normal endogenous spatial and temporal function of ion-conducting proteins during limb regeneration. As we were specifically interested in the role of these signals in pattern formation, rather than wound healing, initiation, and blastema formation (which happen earlier in the regenerative process), we performed our experimental manipulations on blastemas (9 days post amputation) that had already formed and then assessed the consequences of these manipulations when the limb was fully formed at 40 days post amputation.

Overexpressing ion channel proteins Kir2.1, Kv1.5 and NeoNav1.5 causes morphological defects in the regenerated axolotl fore- and hindlimbs

To investigate the function of key ion channels in regeneration, we expressed wild-type or mutant channels to predictably increase or decrease ion flux following blastema formation and then performed skeletal preparations to analyze the skeletal pattern in fully regenerated limbs. To modulate endogenous patterns of membrane potential, we overexpressed the constitutively active inwardly-rectifying Kir2.1-Y242F potassium channel known to hyperpolarize cells; the voltage-gated potassium channel Kv1.5, also known to hyperpolarize cells; or the voltage-gated sodium channel NeoNav1.5, known to depolarize cells. Alterations of membrane potential upon overexpression of these constructs have been previously assessed in various organisms. Miake *et al.* showed that overexpressing Kir2.1 via adenoviral gene transfer in guinea pig cardiac myocytes resulted in increased inwardly rectifying current that regulates cardiac excitability through the control of resting membrane potential assessed by whole-cell patch clamp (Miake *et al.*, 2003). Similarly, Hinard *et al.*, using whole-cell patch-clamp recording, demonstrated that activated Kir2.1 channels are important for human myoblast differentiation (Hinard *et al.*, 2008). In another study by Pai *et al.*, alterations in membrane potential induced by overexpression of Kv1.5 or Nav1.5 in *Xenopus laevis* embryos were demonstrated by voltage reporter dyes such as CC2-DMPE and oocyte clamp techniques. They showed that the electrophysiological state of a cell is important for tissue patterning and that perturbing this state can result in formation of ectopic tissue structures in developing *Xenopus laevis* embryos (Pai *et al.*, 2012b, Pai *et al.*, 2015b). Nav1.5-induced electrophysiological changes were also assessed in cell culture systems. Onkal *et al.* examined the differences between adult and neonatal Nav1.5 isoforms in human embryonic kidney cells using a whole-cell patch clamp technique. They showed that Na+ influx is greater in cells expressing the neonatal Nav1.5 isoform compared to those expressing the adult version and that this difference may have developmental consequences (Onkal *et al.*, 2008). Another study by Fraser *et al.* also showed that the neonatal Nav1.5 isoform is highly expressed in metastatic breast cancer cells and its activity, assessed by patch-clamp techniques, contributes to cellular behaviors, such as increased cellular motility and invasion, that favor the metastatic potential of the cell (Fraser, 2005).

Additionally, to promote the intercellular ion exchange, we overexpressed Cx26, which facilitates formation of constitutively permeable gap junctions between cells. Similar to previous work with ion channels, electrophysiological changes that occur in the cell upon changes to gap junction activity have also been studied in other systems. Connexin mutations have been implicated in deafness and have been shown to influence the conductance of the gap junctions formed by these mutant connexins (White *et al.*, 1998). A work by Richard *et al.* examined the role of a gap junction forming protein Connexin 26 that carries a missense mutation (R75W) in hearing loss. *Xenopus laevis* oocytes expressing Connexin 26 - R75W were shown to fail to induce electrical conductance between cells, as determined by voltage clamp. The failure in conductance was speculated to be caused by improper assembly of the gap junction that interferes with the normal function of gap junctional communication between cells (Richard *et al.*, 1998).

In all of our experiments, expression of EGFP using the same retroviral delivery method served as a control. We initially amputated all four limbs (both fore and hindlimb) of axolotls at the mid-stylopod level. Nine days post-amputation, we transduced blastema cells with retroviruses encoding the aforementioned factors (n>15 animals; 60 limbs per group). The limbs were allowed to regenerate fully and harvested at 40 days post-amputation. Limbs were processed with alcian blue/alizarin red staining to reveal skeletal morphology. Defects were categorized as either major or minor. The major defect category was constituted by examples of digit loss, syndactyly, digit duplication or truncation (Fig. 3 A–J), while the minor defect category was constituted by abnormalities such as defects in carpal morphology (Fig. 3 K–N).

**Fig. 2.**
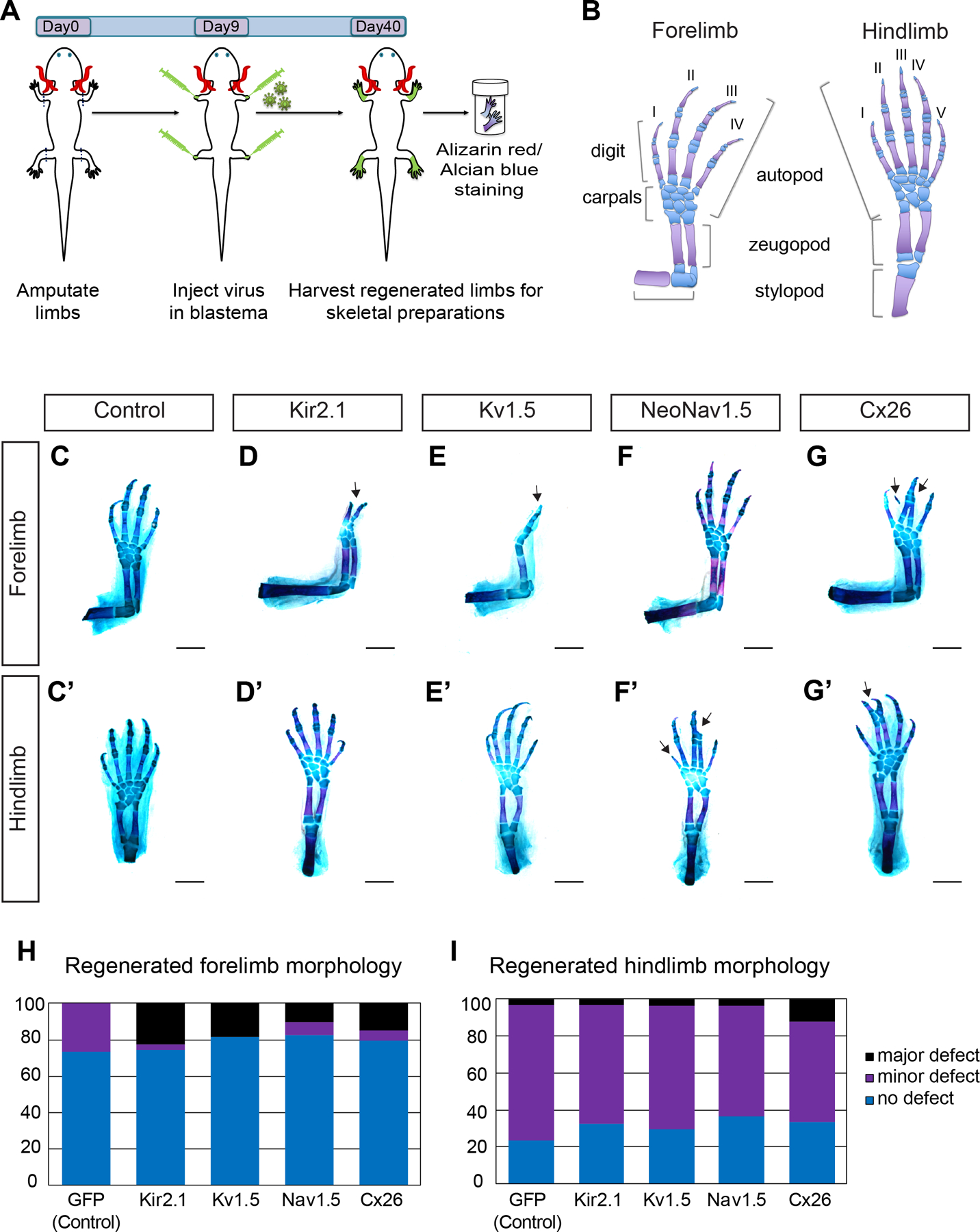
Ion channel and gap junction overexpression causes limb morphology defects in regenerated fore and hindlimbs. **(A)** *Cartoon illustrating the experimental outline. Axolotl fore and hindlimbs were amputated at day 0. 9 days post amputation (Day9) retrovirus carrying ion channel constructs K_ir_2.1, K_v_1.5, NeoNa_v_1.5 and Connexin 26 (Cx26) were injected into blastemas. 40 days post amputation (Day40) all four limbs were harvested and processed with alcian blue (cartilage) and alizarin red (bone) stain for visualization of skeletal morphology*. **(B)** *Cartoon illustrating the fore and hindlimb skeletal elements (cartilage in blue; bone in magenta). Forelimb possess 4 digits and 8 carpals and hindlimb possess 5 digits and 9 tarsals*. **(C-G’)** *Representative regenerated fore* **(C-G)** *and hindlimb* **(C’-G’)** *skeletal morphologies of EGFP control* **(C-C’),** *K_ir_2.1***(D-D’),** *K_v_1.5* **(E-E’),** *NeoNa_v_1.5* **(F-F’)** *and Connexin 26* **(G-G’)** *expressing limbs. Overexpression of ion channel and gap junction protein resulted in major defects elicited as digit* **(D)** *and distal element loss* **(E),** *digit truncation* **(F’)** *and syndactyly* **(G, G’). (H-I)** *Bar graph depicting the quantification of morphological defects observed in ion channel and gap junction overexpressing regenerated limbs. (Scoring details can be found in* Table1). *Scale bar, 1mm*.

In the controls, no major defects were observed in EGFP-overexpressing forelimbs (n=0/34 from 17 animals), while there were minor carpal defects in 26% of the limbs examined (n=9/34). In control hindlimbs, only one major defect was observed in the form of syndactyly (n=1/34 from 17 animals). The majority of the defects we observed were minor defects associated with abnormalities in carpal morphology (n=25/34 limbs from 17 animals) (Fig. 2 C–C’, Table 1). In contrast, we commonly observed major patterning defects following ion channel overexpression. These were most commonly attributable to alterations to the morphology and the number of the regenerated digits. 23% of the K_ir_2.1-Y242F-overexpressing (“OE”) regenerated forelimbs had major defects including digit loss (n=3/31) or digit duplication (n=4/31 limbs) (Fig. 2D, Table 1). Digit loss was observed in only one regenerated hindlimb (n=1/34 limbs). There was only one forelimb with minor carpal morphological defects, while there were 22 hindlimbs with abnormal carpal morphologies, constituting 65% of the K_ir_2.1-Y242F OE hindlimbs examined (n=22/34 limbs from 17 animals, Table 1). 19% of the K_v_1.5 OE forelimbs had major defects in the form of distal element malformation or digit loss (n=2/34 from 17 animals), digit truncation (n=1/34 limbs) or extra digit/spike formation (n=2/34 limbs) (Fig. 2E). K_v_1.5 OE hindlimbs, on the other hand, had only one major defect in the form of digit loss (n=1/27). 67% of the hindlimbs examined had minor defects with fused or split carpal morphology (n=18/27 limbs, Table1). 10% of the NeoNav1.5 OE forelimbs had major defects with digit loss (n=1/29 limbs from 15 animal), digit truncation (n=1/29 limbs) and extra digit formation (n=1/29), while only one hindlimb showed the major defect of syndactyly (n=1/30) (Fig. 2 F–F’). However, 60% of NeoNav1.5 OE hindlimbs had minor carpal morphology defects (n=18/30 limbs), while only 7% of forelimbs had minor defects (n=2/29). We conclude that mis-expression of ion channel proteins in limb blastema cells disturb normal patterning or regenerating limb likely by causing uncoordinated cell behavior that results in morphological defects.

### Overexpressing gap junction protein Cx26 in regenerating limbs causes downstream patterning defects

We performed a similar experiment to investigate how perturbing endogenous patterns of gap junction-mediated cell-cell communication might impact regeneration by using overexpression of Cx26, a truncated Connexin missing its regulatory tail which leads to constitutively permeable gap junction formation (Levin and Mercola, 1998). We found that introduction of this gain-of-function reagent also caused defects in the skeletal morphology of the regenerated limb. In Cx26-overexpressing regenerated forelimbs, 15% had major defects such as loss or malformations of distal elements (n=4/34 limbs from 17 animals) and syndactyly (n=1/34 limbs) and 6% had minor defects with split carpal morphology (n=2/34 limbs) (Fig. 2G, Cx26). Regenerated hindlimbs showed slightly more defects with 12% being major (syndactyly, n=4/33 limbs) and 55 % being minor defects associated with carpal morphology (n=18/33 limbs) (Fig. 2 G–G’). We conclude that precise gap junctional communication is important for normal limb formation and patterning during limb regeneration.

### Acute inhibition of gap junction function by Lindane treatment causes defects in regenerated limbs

Our initial experiments used an overexpression strategy, and we found this caused profound defects in regenerated limb morphology. This treatment, based on known effects on gap junction permeability, may have disrupted the normal pattern and distribution of cell membrane potentials. We also wished to ask if inhibiting the endogenous activities of some of the candidate proteins, and using a different experimental approach, would also impact regenerative outcomes. We therefore used a chemical treatment to inhibit the function of gap junction proteins. We administered lindane in the axolotl housing water over the course of the regeneration (Fig. 4A). Lindane is a γ-hexachlorocyclohexane, and it has been shown that at non-cytotoxic concentrations, it can inhibit gap junction communication (Guan, 1995, Criswell and Lochcaruso, 1995, Li and Mather, 1997) and induce developmental mispatterning via modulation of endogenous gap junction states (Levin and Mercola, 1999, Pai *et al.*, 2015a). We found that administration of lindane at a final concentration of 10mM caused abnormalities in regenerated limbs, which often manifested as loss of carpals and digits or zeugopod malformation (Fig. 4 B–C”). All of the 14 forelimbs examined from seven animals had severe defects compared to DMSO-administered control animals (Fig. 4D), which showed relatively fewer major defects in the form of carpals or digit loss (n=4/14 limbs from 7 animals). These data suggest that communication among cells via gap junctions might hold an important role for the formation of components of the regenerating limb.

## Discussion

Establishment and maintenance of endogenous patterns of specific resting potentials via ion channel- and gap junction-mediated ion flows are critical for setting up physiological boundaries and compartments during development and regeneration (Levin and Martyniuk, 2018, Cervera *et al.*, 2018). They can act by modulating local ion concentration gradients established via the ionic flow mediated by ion channels between the cell and the extracellular environment. Thus, information can be communicated over long distances via diffusion of small molecules and ions through gap junctions formed between cells. This bioelectric gradient established by both local ionic flow and long-range cellular communication activities plays an instructive role in cellular functions like cell viability, proliferation, differentiation, migration or gene expression levels (Sundelacruz *et al.*, 2009) all of which are critical for tissue patterning decisions.

Here, we utilized RNA sequencing data generated from regenerating axolotl limbs over a time course. We investigated the expression dynamics of ion channels and gap junction proteins to determine whether these proteins, which are the contributors to the bioelectric gradient, are involved in regeneration. The transcriptomic analysis showed that a diverse array of ion channel and gap junction isoforms are transcribed in regenerating forelimb tissues, suggesting that these proteins might be participating in regulation of the regeneration process. However, transcription of these genes does not necessarily entirely define the bioelectric state of blastema cells. Bioelectric state is controlled not only by the transcriptional or translational activity of these genes but also by the gating of channels and pumps by their own activity as well as by a range of other physiological and biochemical signals (Pietak and Levin, 2018, Cervera *et al.*, 2018, Pietak and Levin, 2017, Cervera *et al.*, 2020a, Cervera *et al.*, 2019, Cervera *et al.*, 2020b).

We show that challenging ionic homeostasis by overexpressing ion channel proteins Kir2.1 (Y242F), Kv1.5, Nav1.5 or gap junction protein Cx26 resulted in defects in regenerating axolotl limbs which manifested as abnormal distal element formation associated with digit loss, truncation, duplication or syndactyly.

We should also note that the defects associated with ion channel and gap junction mis-expression occurred differentially in forelimb and hindlimbs to some extent. This might be due to the differences in morphogenetic gradients (Diogo *et al.*, 2014) as well as to gene expression patterns employed differentially in forelimbs versus hindlimbs during regeneration (Khan *et al.*, 2002). Additionally, the discrepancy in the number of fore- and hindlimbs examined in given conditions is due to exclusion of images of limbs with low staining or image quality.

Our data show that facilitating gap junction communication via overexpression of Connexin26 within blastema cell populations caused distal element loss or severe malformation and syndactyly in forelimbs and hindlimbs, respectively. On the other hand, interfering with gap junction communication via lindane-induced blockade, which is a general gap junction blocker, caused more severe defects associated with distal elements lost in all limbs examined, indicating the importance of gap junction mediated communication within the blastema cells during regeneration. These data indicate that it is possible that blastema cells must maintain proper membrane potential in order for regeneration to be successful. However, we cannot exclude the possibility that any or all of the factors we modulated might have a separate role, unrelated to regulating cellular membrane potential, and that these role(s) might be crucial for limb regeneration.

Our study, combined with previous studies, proposes a role for precise control of ion channel and gap junction protein activities for proper patterning of limbs during regeneration. This could be linked to the bioelectrical currents and intricate regulation of membrane potential that is effective during the limb regeneration process. Although studies in other systems have shown that misexpression of these ion channel and gap junction proteins alter membrane potential, similar analyses should be performed in regenerating axolotl limbs to further strengthen our hypothesis.

The mechanisms by which membrane potential is necessary for limb regeneration might be at the cellular or the tissue levels. For instance, maintaining ionic homeostasis is critical for controlling cellular functions like cell viability, proliferation, differentiation, migration or gene expression levels. Activity of proteins such as Shh, Fgfs, Bmps, Wnts and Hox, and their precise spatio-temporal expression across fields of cells is important for tissue patterning (Raspopovic *et al.*, 2014, Sheth *et al.*, 2012). Several of these signaling pathways have been shown to be connected to the regulation of membrane potential across many cells generating voltage gradients that can coordinate cellular behavior. For example, Andersen-Tawil syndrome, a rare human disorder that arises due to mutations in K_ir_2.1 channels, causes morphological defects including craniofacial dysmorphologies as well as clinodactyly (permanent curvature of a finger or toe) or syndactyly (webbing between finger) (Plaster *et al.*, 2001). Adams *et al.*, studied the role of Kir2.1 mis-expression in *Xenopus laevis* embryos and showed that ectopic expression of Kir2.1 wildtype or ATS associated variants results in craniofacial defects and causes changes in the expression pattern of key neural crest markers that are important for the normal patterning of the face (Adams *et al.*, 2016b).

Mis-regulated K_ir_2.1 expression contributing to morphological defects has been observed during mouse digit and fly wing patterning and growth (Dahal *et al.*, 2012) as well. Dahal *et al.* also showed that defects associated with *Drosophila* wing patterning that arose due to K_ir_2.1 misregulation were similar to phenotypes resultant from disruption of the TGFb/ BMP signaling pathway. They identified that perturbed K_ir_2.1 channel function was causing the reduction of Dpp (*Drosophila* ortholog of Bmp) signaling and associated wing morphology defects (Dahal *et al.*, 2017).

In addition to morphogenesis, K_ir_2.1 has been implicated in osteogenesis and chondrogenesis through the Bmp/SMAD/RUNX2 pathway in cultured mesenchymal stromal cells (Pini *et al.*, 2018). Several other potassium channels are also implicated in human developmental disorders associated with craniofacial and digital morphological abnormalities (George *et al.*, 2019). The regulation between ionic state and limb patterning gene expression can be regulated in the opposite direction as well. For example, Shh and Wnt signaling were shown to promote gap junctional communication via activating *connexin43* expression, which was required to synchronize Ca^++^ oscillations to coordinate feather mesenchymal cell migration necessary for chicken feather morphogenesis (Li A. *et al.*, 2018). Additionally, gap junctions Cx43 and Cx32 expression in the posterior developing chick and mouse limb bud shown to be sensitive to FGF4 (Makarenkova *et al.*, 1997).

Developmental signaling pathways are activated during regeneration process to instruct blastema cells to reform the lost tissue (Nacu and Tanaka, 2011). Therefore, future work is needed to examine the expression profile of these developmental digit patterning genes in hyperpolarized/depolarized regenerating limbs. It is also worth investigating cellular behaviors such as proliferation, differentiation and migration as potential results of the interplay between bioelectric state of the cell and morphogen gradients established during patterning. Finally, the future development of transgenic axolotls expressing genetically-encoded voltage indicator proteins (St-Pierre *et al.*, 2015) will be important tools for addressing these questions. These approaches may include ArcLight (Jin. *et al.*, 2012), which exhibit fluorescent intensity changes in response to voltage fluctuations as well as calcium-dependent indicators like CAMPARI, in which green-to-red fluorescence conversion occurs when elevated Ca^2+^ levels meet with the experimenter-controlled illumination (Fosque *et al.*, 2015). These tools will enable real-time monitoring of spatio-temporal bioelectrical gradients and their outcomes during limb regeneration. They can also be integrated with other genetic tools that report on other aspects of cell state.

In summary, our results indicate that once a blastema has formed, the cells within it may use precise modulation of bioelectric state at the spatial and temporal level as a means to orchestrate pattern formation in the regenerated limb. Perturbing the endogenous mechanisms for establishing and executing this pattern can lead to defects in the final morphologies of the regenerated limbs. While future work is necessary to elucidate the exact mechanism of how these morphological defects arose due to mis-expression of ion channels and gap junction proteins, involvement of membrane potential in orchestrating regenerating axolotl limb morphology provides the basis for future studies to make the connection between the patterning and bioelectrical state of the regenerating limb.

## Materials and Methods

### Animal handling and procedures

All animal experiments were performed according to IACUC protocol #2016N000369 at Brigham and Women’s Hospital and IACUC protocol #19-02-346 at Harvard University. Leucistic axolotls were used throughout the study and maintained as described previously (Bryant *et al.*, 2017). Animals were anesthetized in 0.1% tricaine during limb amputation procedures. All limb amputations were conducted at the mid-stylopod level.

Following amputation, the bone was trimmed to allow for wound epidermis formation. Animals were allowed to recover in 0.5% sulfamerazine overnight and transferred to regular axolotl housing water until the end of the study. Regenerated limbs were harvested 10 weeks after the amputation. To harvest limbs, animals were anesthetized in 0.1% tricane, and harvested limbs were processed for skeletal staining.

### Expression analysis

RNA-seq. data from Stewart *et al.*, 2013 was used to examine the expression of transcripts encoding sodium and potassium channels as well as the connexin gap junction proteins in intact (homeostasis) and regenerating forelimbtissuesamplesattimepoints;3h,6h,12h,1d,3d,5d,7d,10 d, 14 d, 21 d, 28 d. We only examined genes with expression levels higher than 1 (TPM >1) at homeostasis (0 h). Expression of transcripts encoding potassium channels, sodium channels, and gap junction proteins are represented in the heat map by computing the the log2 ratios of TPMs (transcripts per million) of each time point relative to the time-zero control (Fig. 1C).

### Virus production and injection

Viruses were produced as described previously (Whited *et al.*, 2013). For each 10 cm dish, 6 mg of constructs expressing human gap junction protein Connexin26 (pQC-Cx26-2A-mCherry) or human ion channel coding proteins (pQC-EGFP, pQC-Kv1.5–2A-EGFP (subcloned from (Strutz-Seebohm *et al.*, 2007)), pQC-Kir2.1(Y242F)-2A-EGFP (original KCNJ2(Kir2.1)-Y242F construct was received from S.Konig and L.Bernheim (Hinard *et al.*, 2008) by Levin lab and subcloned into pQC vector in the Whited lab), pQC-NeoNav1.5-2A-EGFP (neonatal isoform of Nav1.5 subcloned from (Chioni *et al.*, 2005) were mixed with 3 mg helper (encoding gag/pol) and 1mg encoding coat protein (vsvg) along with 35 ml Invitrogen p3000 transfection reagent in 500 ml Opti-MEM (GIBCO). In another tube 41 ml of Invitrogen Lipofectamine p3000 reagent was mixed with 500 ml opti-MEM (GIBCO) and then the two solutions were mixed together and left to sit at room temperature for 15 min. For each construct, 12 plates of HEK293 cells at a confluency of 80% were used to transfect for virus production. Cell culture media was replaced with opti-MEM and 1100 ml of transfection mixture was added into the culture media. Cells were kept in transfection media overnight. Transfection media was replaced with DMEM containing 10%Nu-serum and 1% Pen/Strep the next day. Starting on the third day (2 days post transfection) media containing virus was collected into 50 ml canonical tubes and stored at −80°C. Collections were repeated for 4 days, which yielded a total of 480 ml of media loaded with virus. Collected virus-media suspension was stored at −80°C until the virus concentration step.

In order to concentrate the virus, collected media containing the virus was thawed and filtered through Centricon Plus-70 filters (Millipore) followed by ultracentrifugation at 20K for 1 h 30 min at 4°C. The supernatant was discarded as much as possible and the remaining virus mix was stored at 4°C overnight. The virus mix was aliquoted into tubes the next day sparing 1.2 ml to be used for titering. In order to titer the virus, HEK293 cells from a nearly confluent 10 cm dish were split into 6-well plate with 1:20 dilution. A serial dilution of virus from 1:10^3^ to 1:10^8^ was prepared. 1.2 ml of virus suspension was added into 1200 ml of media (1:10^3^) and serially diluted from there to 1:10^8^. Virus containing media was added into the cells in 6-well plate and transduction efficiency was imaged the following day. The number of fluorescent cells was counted as an indication of transduction. The concentration of stock was calculated based on the number of the virus counted and the dilution factor.

### Skeletal preparations

**Fig. 3.**
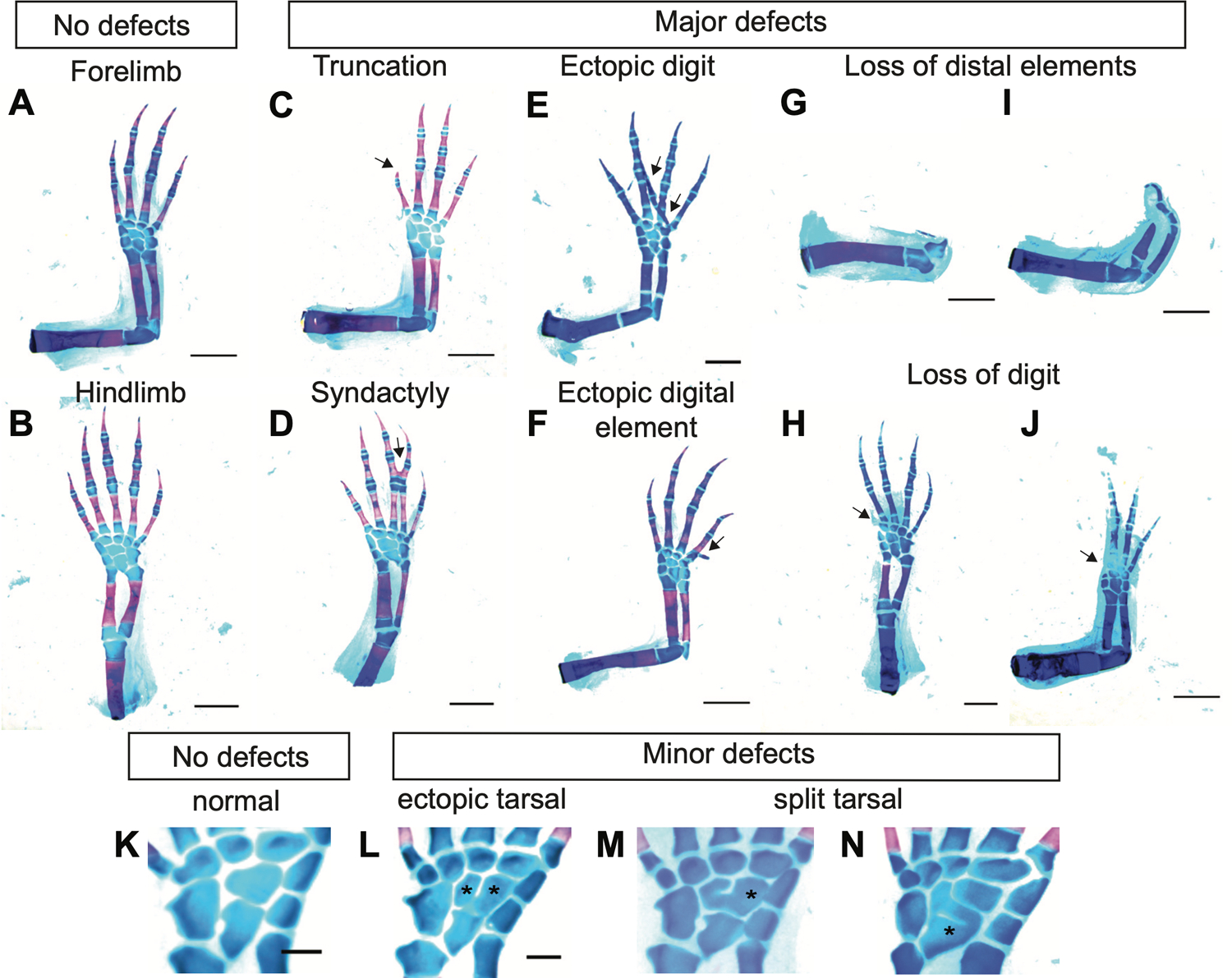
Representative alcian blue/ alizarin red images depicting skeletal morphology defects used in scoring. **(A,B)** *Representative fore and hindlimbs with no defect. Representative images of major defects, truncation* **(C)**, *syndactyly* **(D)**, *ectopic digit formation* **(E)**, *ectopic digital element formation* **(F)**, *loss of distal elements* **(G-I)** *digit loss* **(H-J)***. Scale bar, 2 mm*. **(K-N)** *Representative images of central elements* **(K)** *normal central elements present in hindlimb with 9 tarsals*. **(L)** *ectopic tarsal,* **(M,N)** *split tarsal. Scale bar, 0.5 mm*.

**Fig. 4.**
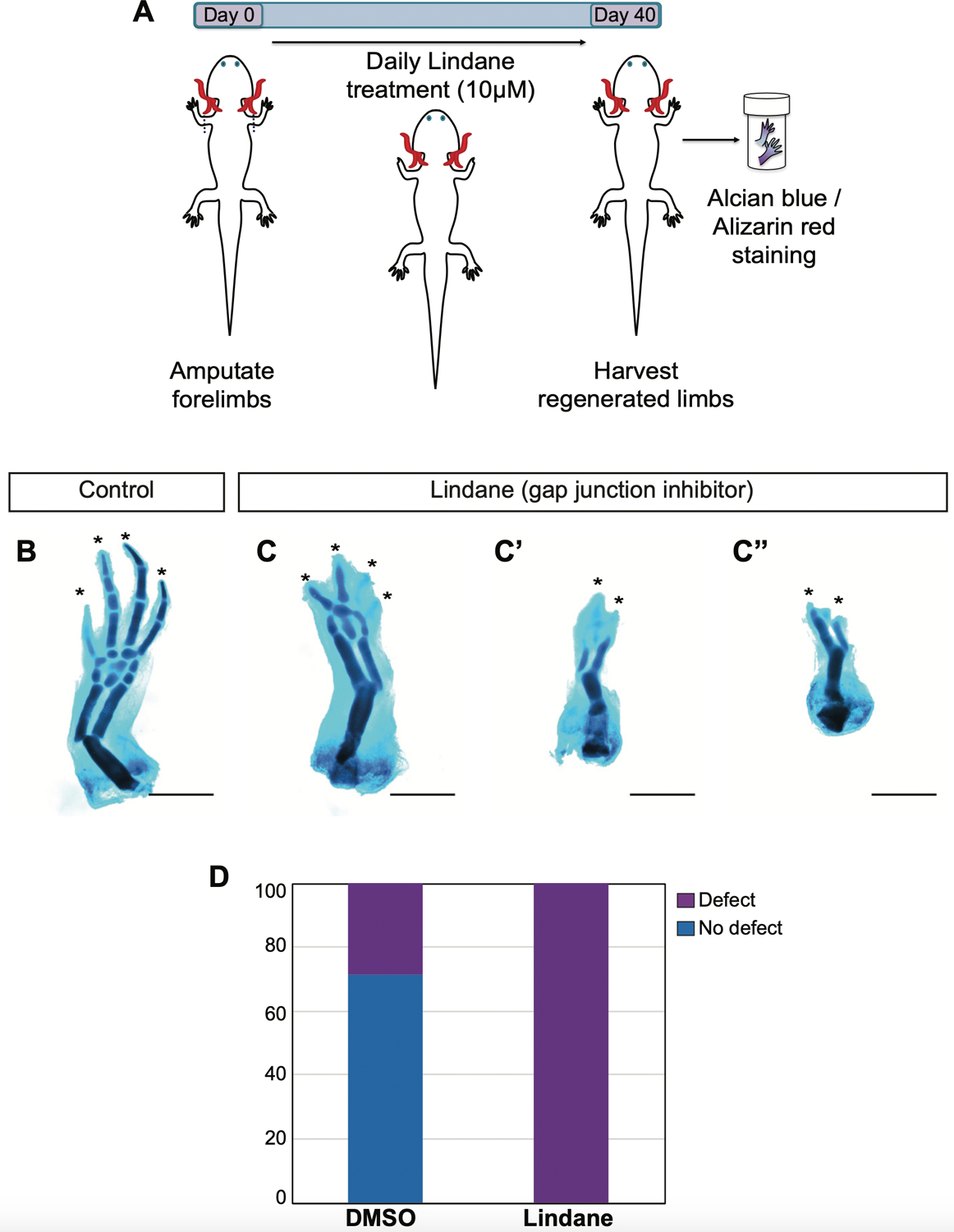
The gap junction blocker lindane causes severe skeletal morphological defects in regenerating forelimbs. **(A)** *Cartoon illustrating the experimental timeline. Axolotl forelimbs were amputated at day 0. Lindane at a final concentration of 10* m*M is added daily into axolotl housing water until the limbs are harvested 40 days after amputation. Harvested limbs are processed for skeletal morphology evaluation*. **(B-C”)** *Representative skeletal images of DMSO* **(B)** *and lindane* **(C,C”)** *treated control and gap junction communication blocked forelimbs*. **(D)** *Bar graph depicting the scoring of the defects observed in DMSO treated control (4 out of 14 limb showed defect) versus lindane treated forelimbs (all 14 forelimbs examined showed defect). Scale bar, 1 mm*.

To analyze the skeletal pattern, regenerated limbs were stained with Alcian blue/ Alizarin red as described previously (Whited *et al.*, 2012). Limbs were fixed in 95% ethanol overnight at room temperature followed by 100% acetone incubation under the same conditions. Samples were then transferred into Alcian blue/Alizarin red solution. Samples were then cleared in a 1% hydroxide solution and stored in glycerol. Images were captured using a Leica M165 FC equipped with Leica DFC310 FX camera. Skeletal morphology defects associated with digit loss, syndactyly, polydactyly, extra digital element formation or more severe distal element loss or malformation were classified as major, while defects associated with carpal/tarsal formation considered as minor (Fig. 3 and Tables 2 –3).

### Lindane treatment

For acute inhibition of gap junction formation, Lindane reconstituted in DMSO to a final concentration of 10 mM was administered into the axolotl housing water to a final concentration of 10 mM. Lindane administration was repeated daily by full water change over the course of the regeneration for 40 days. As a control DMSO was administered at a 1:1000 ratio.

## Supplementary Material

Supplementary Tables 1 and 2 **Supplementary Table 1. Forelimb morphological outcomes following ion channel / gap junction overexpression.** Table listing the morphological defects observed with each forelimb sample examined in GFP (control), K_ir_2.1, K_v_1.5, NeoNa_v_1.5 and Connexin 26 (Cx26).
**Supplementary Table 2. Hindlimb morphological outcomes following ion channel / gap junction overexpression** Table listing the morphological defects observed with each hindlimb sample examined in GFP (control), K_ir_2.1, K_v_1.5, NeoNa_v_1.5 and Connexin 26 (Cx26).

## Figures and Tables

**Table 1.** Morphological defect scoring in regenerated limbs. Major defects are classified as digit/ distal element loss, syndactyly, truncated digit and extra digit/spike formation. Minor defects are classified as carpal/tarsal morphology defects associated with ectopic central element formation, split or fusion.

			Major defects				Minor defects	Total limbs p-value
Condition	Limb	No defect	Digit Loss	Syndactyly	Truncated digit	Extra digit/spike	Carpal/tarsal defects	
Control (EGFP)	Forelimb	25	0	0	0	0	9	34
	Hindlimb	8	0	1	0	0	25	34
Cx26	Forelimb	27	3	1	0	1	2	34 p=0.006 **
	Hindlimb	11	0	4	0	0	18	33 p=0.185 ns
Kir2.1	Forelimb	23	3	0	0	4	1	31 p=0.001 ***
	Hindlimb	11	1	0	0	0	22	34 p=0.712 ns
Kv1.5	Forelimb	22	2	0	1	2	0	27 p=0.0003 ***
	Hindlimb	14	0	0	0	0	20	27 p=0.884 ns
Nav1.5	Forelimb	24	1	0	1	1	2	29 p=0.028 *
	Hindlimb	13	0	1	0	0	16	30 p=0.576 ns

Fisher’s exact tests with 2×3 contingency table (control vs treated) were applied to calculate p-values.

* p<0.05,

** p<0.01,

*** p<0.001.

## Abbreviations used in this paper:

Bmp: bone morphogenic protein
Cx26: connexin26
EGFP: enhanced green fluorescent protein
Fgf: fibroblast growth factor
Kir2.1: inward-rectifier potassium ion channel
Kv1.5: voltage-gated potassium channel
NeoNav1.5: a neonatal isoform of voltage-gated sodium channel, Shh, sonic hedgehog
TPM: transcripts per million
Vmem: membrane voltage potential
Wnt: wingless-related integration site
