# Supplementary material for: Precise control of ion channel and gap junction expression is required for patterning of the regenerating axolotl limb
Source: Int J Dev Biol. Author manuscript; Available in PMC 2022 Jan 28. (PMC8796139; doi:10.1387/ijdb.200114jw)
Supplement: Supplementary Tables 1 and 2 — Supplementary Table 1. Forelimb morphological outcomes following ion channel / gap junction overexpression. Table listing the morphological defects observed with each forelimb sample examined in GFP (control), Kir2.1, Kv1.5, NeoNav1.5 and Connexin 26 (Cx26). Supplementary Table 2. Hindlimb morphological outcomes following ion channel / gap junction overexpression Table listing the morphological defects observed with each hindlimb sample examined in GFP (control), Kir2.1, Kv1.5, NeoNav1.5 and Connexin 26 (Cx26). [file NIHMS1709191-supplement-Supplementary_Tables_1_and_2.pdf]

Supplementary Table 2: Forelimb morphological outcomes following ion channel / gap junction overexpression

**Forelimb sample number**	**Classification**	**Description**
GFP control 1.1	No defect	Normal
GFP control 1.2	No defect	Normal
GFP control 2.1	No defect	Normal
GFP control 2.2	No defect	Normal
GFP control 3.1	No defect	Normal
GFP control 3.2	No defect	Normal
GFP control 4.1	Minor	Ectopic carpal formation
GFP control 4.2	No defect	Normal
GFP control 5.1	No defect	Normal
GFP control 5.2	No defect	Normal
GFP control 6.1	Minor	Ectopic carpal formation
GFP control 6.2	Minor	Ectopic carpal formation
GFP control 7.1	No defect	Normal
GFP control 7.2	No defect	Normal
GFP control 8.1	No defect	Normal
GFP control 8.2	Minor	Ectopic carpal formation
GFP control 9.1	No defect	Normal
GFP control 9.2	No defect	Normal
GFP control 10.1	Minor	Ectopic carpal formation
GFP control 10.2	No defect	Normal
GFP control 11.1	Minor	Ectopic carpal formation
GFP control 11.2	Minor	Ectopic carpal formation
GFP control 12.1	Minor	Ectopic carpal formation
GFP control 12.2	No defect	Normal
GFP control 13.1	No defect	Normal
GFP control 13.2	No defect	Normal
GFP control 14.1	No defect	Normal
GFP control 14.2	No defect	Normal
GFP control 15.1	No defect	Normal
GFP control 15.2	No defect	Normal
GFP control 16.1	No defect	Normal
GFP control 16.2	No defect	Normal
GFP control 17.1	No defect	Normal
GFP control 17.2	Minor	Ectopic carpal formation

Forelimb sample number	Classification	Description
CX26 1.1	No defect	Normal
CX26 1.2	Minor	Carpal fusion
CX26 2.1	No defect	Normal
CX26 2.2	Minor	Ectopic carpal formation
CX26 3.1	No defect	Normal
CX26 3.2	Major	Syndactyly between digit 2-3; ectopic digital element growth
CX26 4.1	No defect	Normal
CX26 4.2	No defect	Normal
CX26 5.1	No defect	Normal
CX26 5.2	No defect	Normal
CX26 6.1	No defect	Normal
CX26 6.2	Major	Ectopic digital element growth
CX26 7.1	No defect	Normal
CX26 7.2	No defect	Normal
CX26 8.1	Major	Loss of carpals and digits
CX26 8.2	Major	Loss of zeugopod and autopod
CX26 9.1	No defect	Normal
CX26 9.2	No defect	Normal
CX26 10.1	No defect	Normal
CX26 10.2	Major	Loss of digit 1, digit 2 and 3 fusion and truncation, digit 4 truncation
CX26 11.1	No defect	Normal
CX26 11.2	No defect	Normal
CX26 12.1	No defect	Normal
CX26 12.2	No defect	Normal
CX26 13.1	No defect	Normal
CX26 13.2	No defect	Normal
CX26 14.1	No defect	Normal
CX26 14.2	No defect	Normal
CX26 15.1	No defect	Normal
CX26 15.2	No defect	Normal
CX26 16.1	No defect	Normal
CX26 16.2	No defect	Normal
CX26 17.1	No defect	Normal
CX26 17.2	No defect	Normal

**Forelimb sample number**	**Classification**	**Description**
Kir2.1 (Y242F) 1.1	No defect	Normal
Kir2.1 (Y242F) 1.2	No defect	Normal
Kir2.1 (Y242F) 2.1	No defect	Normal
Kir2.1 (Y242F) 2.2	No defect	Normal
Kir2.1 (Y242F) 3.1	Major	Loss of several carpals and 2 digits. Formed digits are truncated
Kir2.1 (Y242F) 3.2	Major	Digit malformation and truncation
Kir2.1 (Y242F) 4.1	No defect	Normal
Kir2.1 (Y242F) 4.2	No defect	Normal
Kir2.1 (Y242F) 5.1	No defect	Normal
Kir2.1 (Y242F) 5.2	No defect	Normal
Kir2.1 (Y242F) 6.1	No defect	Normal
Kir2.1 (Y242F) 6.2	No defect	Normal
Kir2.1 (Y242F) 7.1	Major	Ectopic digital element growth
Kir2.1 (Y242F) 7.2	No defect	Normal
Kir2.1 (Y242F) 8.1	No defect	Normal
Kir2.1 (Y242F) 8.2	No defect	Normal
Kir2.1 (Y242F) 9.1	No defect	Normal
Kir2.1 (Y242F) 9.2	Major	Truncation in digit 3; bifurcation of digit 4, ectopic digital element formation from ulna
Kir2.1 (Y242F) 10.1	Major	Truncation in digit 3; bifurcation of digit 4
Kir2.1 (Y242F) 10.2	No defect	Normal
Kir2.1 (Y242F) 11.1	No defect	Normal
Kir2.1 (Y242F) 11.2	No defect	Normal
Kir2.1 (Y242F) 12.1	Exclude	Low staining or imaging quality
Kir2.1 (Y242F) 12.2	Major	Loss of digit 1
Kir2.1 (Y242F) 13.1	No defect	Normal
Kir2.1 (Y242F) 13.2	No defect	Normal
Kir2.1 (Y242F) 14.1	No defect	Normal
Kir2.1 (Y242F) 14.2	No defect	Normal
Kir2.1 (Y242F) 15.1	Exclude	Low staining or imaging quality
Kir2.1 (Y242F) 15.2	No defect	Normal
Kir2.1 (Y242F) 16.1	Exclude	Low staining or imaging quality
Kir2.1 (Y242F) 16.2	Major	Truncation in digit 1; spike formation from digit 4
Kir2.1 (Y242F) 17.1	Minor	Ectopic carpal formation
Kir2.1 (Y242F) 17.2	No defect	Normal

Forelimb sample number	Classification	Description
Kv1.5 1.1	No defect	Normal
Kv1.5 1.2	No defect	Normal
Kv1.5 2.1	Exclude	Low staining or imaging quality
Kv1.5 2.2	Exclude	Low staining or imaging quality
Kv1.5 3.1	No defect	Normal
Kv1.5 3.2	No defect	Normal
Kv1.5 4.1	No defect	Normal
Kv1.5 4.2	No defect	Normal
Kv1.5 5.1	No defect	Normal
Kv1.5 5.2	No defect	Normal
Kv1.5 6.1	No defect	Normal
Kv1.5 6.2	No defect	Normal
Kv1.5 7.1	Major	Truncation in digit 1
Kv1.5 7.2	No defect	Normal
Kv1.5 8.1	No defect	Normal
Kv1.5 8.2	No defect	Normal
Kv1.5 9.1	Exclude	Low staining or imaging quality
Kv1.5 9.2	Major	Polydactyly
Kv1.5 10.1	Exclude	Low staining or imaging quality
Kv1.5 10.2	Exclude	Low staining or imaging quality
Kv1.5 11.1	No defect	Normal
Kv1.5 11.2	Exclude	Low staining or imaging quality
Kv1.5 12.1	No defect	Normal
Kv1.5 12.2	Major	Polydactyly
Kv1.5 13.1	No defect	Normal
Kv1.5 13.2	No defect	Normal
Kv1.5 14.1	No defect	Normal
Kv1.5 14.2	No defect	Normal
Kv1.5 15.1	No defect	Normal
Kv1.5 15.2	No defect	Normal
Kv1.5 16.1	Major	Loss of radius; loss of carpals; loss of digits 1-3; truncation of digit 4
Kv1.5 16.2	Major	Loss of radius; loss of carpals; loss of digits 1-3; truncation of digit 4
Kv1.5 17.1	Exclude	Low staining or imaging quality
Kv1.5 17.2	No defect	Normal

Forelimb sample number	Classification	Description
Nav1.5 1.1	No defect	Normal
Nav1.5 1.2	Minor	Ectopic carpal formation
Nav1.5 2.1	No defect	Normal
Nav1.5 2.2	No defect	Normal
Nav1.5 3.1	No defect	Normal
Nav1.5 3.2	No defect	Normal
Nav1.5 4.1	Major	Truncation in digit 1
Nav1.5 4.2	No defect	Normal
Nav1.5 5.1	No defect	Normal
Nav1.5 5.2	Major	Loss of digit 4
Nav1.5 6.1	No defect	Normal
Nav1.5 6.2	Exclude	low staining or imaging quality
Nav1.5 7.1	No defect	Normal
Nav1.5 7.2	Minor	Carpal fusion
Nav1.5 8.1	No defect	Normal
Nav1.5 8.2	No defect	Normal
Nav1.5 9.1	No defect	Normal
Nav1.5 9.2	No defect	Normal
Nav1.5 10.1	No defect	Normal
Nav1.5 10.2	No defect	Normal
Nav1.5 11.1	No defect	Normal
Nav1.5 11.2	Major	Polydactyly
Nav1.5 12.1	No defect	Normal
Nav1.5 12.2	No defect	Normal
Nav1.5 13.1	No defect	Normal
Nav1.5 13.2	No defect	Normal
Nav1.5 14.1	No defect	Normal
Nav1.5 14.2	No defect	Normal
Nav1.5 15.1	No defect	Normal
Nav1.5 15.2	No defect	Normal

Supplementary Table 3: Hindlimb morphological outcomes following ion channel / gap junction overexpression

Hindlimb sample number	Classification	Description
GFP control 1.1	No defect	Normal
GFP control 1.2	No defect	Normal
GFP control 2.1	Minor	Ectopic tarsal formation
GFP control 2.2	No defect	Normal
GFP control 3.1	Minor	Ectopic tarsal formation
GFP control 3.2	Minor	Ectopic tarsal formation
GFP control 4.1	No defect	Normal
GFP control 4.2	Minor	Ectopic tarsal formation
GFP control 5.1	Minor	Ectopic tarsal formation
GFP control 5.2	Minor	Ectopic tarsal formation
GFP control 6.1	Minor	Ectopic tarsal formation
GFP control 6.2	Minor	Ectopic tarsal formation
GFP control 7.1	Minor	Ectopic tarsal formation
GFP control 7.2	Minor	Ectopic tarsal formation
GFP control 8.1	Minor	Ectopic tarsal formation
GFP control 8.2	Minor	Ectopic tarsal formation
GFP control 9.1	Minor	Ectopic tarsal formation
GFP control 9.2	Minor	Ectopic tarsal formation
GFP control 10.1	Minor	Ectopic tarsal formation
GFP control 10.2	Minor	Ectopic tarsal formation
GFP control 11.1	Minor	Ectopic tarsal formation
GFP control 11.2	Minor	Ectopic tarsal formation
GFP control 12.1	No defect	Normal
GFP control 12.2	Minor	Ectopic tarsal formation
GFP control 13.1	Minor	Ectopic tarsal formation
GFP control 13.2	Minor	Ectopic tarsal formation
GFP control 14.1	Minor	Ectopic tarsal formation
GFP control 14.2	Minor	Ectopic tarsal formation
GFP control 15.1	Minor	Ectopic tarsal formation
GFP control 15.2	No defect	Normal
GFP control 16.1	No defect	Normal
GFP control 16.2	No defect	Normal
GFP control 17.1	Major	Syndactyly between digit 2 and 3
GFP control 17.2	Minor	Ectopic tarsal formations

Hindlimb sample number	Classification	Description
CX26 1.1	Minor	Ectopic tarsal formations
CX26 1.2	No defect	Normal
CX26 2.1	Minor	Ectopic tarsal formations
CX26 2.2	Minor	Ectopic tarsal formations
CX26 3.1	Major	Syndactyly between digit 2-3; ectopic tarsal formation
CX26 3.2	Minor	Ectopic tarsal formation
CX26 4.1	No defect	Normal
CX26 4.2	No defect	Normal
CX26 5.1	Minor	Hole in middle tarsal
CX26 5.2	Minor	Hole in middle tarsal
CX26 6.1	Minor	Ectopic tarsal formation
CX26 6.2	Minor	Split tarsal formation
CX26 7.1	Major	Syndactyly between digit 2-3
CX26 7.2	No defect	Normal
CX26 8.1	No defect	Normal
CX26 8.2	Minor	Loss of tarsal
CX26 9.1	Minor	Ectopic tarsal formations
CX26 9.2	Minor	Ectopic tarsal formations
CX26 10.1	No defect	Normal
CX26 10.2	No defect	Normal
CX26 11.1	Exclude	low staining or imaging quality
CX26 11.2	Minor	Split tarsal formation
CX26 12.1	Minor	Ectopic tarsal formations
CX26 12.2	Minor	Several ectopic tarsal formations
CX26 13.1	Major	Syndactyly between digit 2-3; split tarsal formation
CX26 13.2	Major	Syndactyly between digit 2-3; split tarsal formation
CX26 14.1	No defect	Normal
CX26 14.2	No defect	Normal
CX26 15.1	Minor	Tarsal fusion
CX26 15.2	Minor	Tarsal fusion
CX26 16.1	Minor	Hole in middle tarsal
CX26 16.2	Minor	Hole in middle tarsal
CX26 17.1	No defect	Normal
CX26 17.2	No defect	Normal

Hindlimb sample number	Classification	Description
Kir2.1 (Y242F) 1.1	No defect	Normal
Kir2.1 (Y242F) 1.2	Minor	Ectopic tarsal formation
Kir2.1 (Y242F) 2.1	Minor	Tarsal protrusion
Kir2.1 (Y242F) 2.2	Minor	Ectopic tarsal formation
Kir2.1 (Y242F) 3.1	Minor	Tarsal fusion
Kir2.1 (Y242F) 3.2	Minor	Tarsal fusion
Kir2.1 (Y242F) 4.1	Minor	Ectopic tarsal formation
Kir2.1 (Y242F) 4.2	No defect	Normal
Kir2.1 (Y242F) 5.1	Minor	Ectopic tarsal formation
Kir2.1 (Y242F) 5.2	Major	Loss of digit 1
Kir2.1 (Y242F) 6.1	Minor	Ectopic tarsal formation
Kir2.1 (Y242F) 6.2	No defect	Normal
Kir2.1 (Y242F) 7.1	No defect	Normal
Kir2.1 (Y242F) 7.2	No defect	Normal
Kir2.1 (Y242F) 8.1	Minor	Ectopic tarsal formation
Kir2.1 (Y242F) 8.2	No defect	Normal
Kir2.1 (Y242F) 9.1	No defect	Normal
Kir2.1 (Y242F) 9.2	Minor	Ectopic tarsal formation
Kir2.1 (Y242F) 10.1	Minor	Ectopic tarsal formation
Kir2.1 (Y242F) 10.2	No defect	Normal
Kir2.1 (Y242F) 11.1	Minor	Ectopic tarsal formation and fusion
Kir2.1 (Y242F) 11.2	Minor	Ectopic tarsal formation
Kir2.1 (Y242F) 12.1	No defect	Normal
Kir2.1 (Y242F) 12.2	Minor	Ectopic tarsal formation
Kir2.1 (Y242F) 13.1	Minor	Tarsal protrusion
Kir2.1 (Y242F) 13.2	Minor	Ectopic tarsal formation
Kir2.1 (Y242F) 14.1	Minor	Ectopic tarsal formation
Kir2.1 (Y242F) 14.2	No defect	Normal
Kir2.1 (Y242F) 15.1	Minor	Hole in middle tarsal
Kir2.1 (Y242F) 15.2	Minor	Hole in middle tarsal
Kir2.1 (Y242F) 16.1	No defect	Normal
Kir2.1 (Y242F) 16.2	Minor	Split middle tarsal
Kir2.1 (Y242F) 17.1	Minor	Split middle tarsal
Kir2.1 (Y242F) 17.2	Minor	Ectopic carpal formation

Hindlimb sample number	Classification	Description
Kv1.5 1.1	No defect	Normal
Kv1.5 1.2	No defect	Normal
Kv1.5 2.1	Minor	Slit in middle tarsal
Kv1.5 2.2	Major	Loss of digit 4
Kv1.5 3.1	Minor	Ectopic tarsal formation
Kv1.5 3.2	Minor	Ectopic tarsal formation
Kv1.5 4.1	Minor	Ectopic tarsal formation
Kv1.5 4.2	No defect	Normal
Kv1.5 5.1	Minor	Ectopic tarsal formation
Kv1.5 5.2	No defect	Normal
Kv1.5 6.1	Exclude	low staining or imaging quality
Kv1.5 6.2	Minor	Slit in middle tarsal
Kv1.5 7.1	Minor	Ectopic tarsal formation
Kv1.5 7.2	Minor	Ectopic tarsal formation
Kv1.5 8.1	Minor	Ectopic tarsal formation
Kv1.5 8.2	Minor	Ectopic tarsal formation
Kv1.5 9.1	Minor	Ectopic tarsal formation
Kv1.5 9.2	Minor	Ectopic tarsal formation; knuckle fusion in digit 4
Kv1.5 10.1	Exclude	low staining or imaging quality
Kv1.5 10.2	Minor	Ectopic tarsal formation
Kv1.5 11.1	Exclude	low staining or imaging quality
Kv1.5 11.2	Exclude	low staining or imaging quality
Kv1.5 12.1	Minor	Ectopic tarsal formation
Kv1.5 12.2	Minor	Ectopic tarsal formation
Kv1.5 13.1	Exclude	low staining or imaging quality
Kv1.5 13.2	Exclude	low staining or imaging quality
Kv1.5 14.1	No defect	Normal
Kv1.5 14.2	No defect	Normal
Kv1.5 15.1	Minor	Ectopic tarsal formation
Kv1.5 15.2	No defect	Normal
Kv1.5 16.1	Minor	Slit in middle tarsal
Kv1.5 16.2	Minor	Ectopic tarsal formation
Kv1.5 17.1	Exclude	low staining or imaging quality
Kv1.5 17.2	No defect	Normal

Forelimb sample number	Classification	Description
Nav1.5 1.1	Minor	Slit in middle tarsal
Nav1.5 1.2	Minor	Slit in middle tarsal
Nav1.5 2.1	Minor	Slit in middle tarsal
Nav1.5 2.2	Minor	Slit in middle tarsal
Nav1.5 3.1	No defect	Normal
Nav1.5 3.2	Minor	Ectopic tarsal formation
Nav1.5 4.1	Minor	Ectopic tarsal formation
Nav1.5 4.2	Minor	Ectopic tarsal formation
Nav1.5 5.1	Minor	Ectopic tarsal formation
Nav1.5 5.2	Minor	Ectopic tarsal formation
Nav1.5 6.1	Minor	Ectopic tarsal formation
Nav1.5 6.2	No defect	Normal
Nav1.5 7.1	Minor	Slit in middle tarsal
Nav1.5 7.2	No defect	Normal
Nav1.5 8.1	Minor	Ectopic tarsal formation
Nav1.5 8.2	Major	Truncation in digit 1; syndactyly between digit 3 and 4
Nav1.5 9.1	No defect	Normal
Nav1.5 9.2	No defect	Normal
Nav1.5 10.1	No defect	Normal
Nav1.5 10.2	No defect	Normal
Nav1.5 11.1	No defect	Normal
Nav1.5 11.2	Minor	Tarsal protrusion
Nav1.5 12.1	Minor	Slit in middle tarsal
Nav1.5 12.2	No defect	Normal
Nav1.5 13.1	Minor	Ectopic tarsal formation
Nav1.5 13.2	Minor	Slit in middle tarsal
Nav1.5 14.1	No defect	Normal
Nav1.5 14.2	Minor	Ectopic tarsal formation
Nav1.5 15.1	No defect	Normal
Nav1.5 15.2	Minor	Ectopic tarsal formation
